# PD238 Evolution Of The Consumer Evidence And Engagement Unit And Contribution To Consumer Input In Australian Health Technology Assessment Processes

**DOI:** 10.1017/S0266462324004550

**Published:** 2025-01-07

**Authors:** Jo Watson, Rebecca Trowman

## Abstract

**Introduction:**

Various committees provide health technology assessment (HTA) recommendations to the Australian government on health technology subsidization. All committees include consumer members, but operations vary across committees. To support the committee’s consumer members and facilitate consumer input, the Consumer Evidence and Engagement Unit (CEEU) was launched in 2019. The CEEU aims to enhance inclusion of consumer input in Commonwealth HTA processes.

**Methods:**

The CEEU operates within the Australian Government Department of Health and Aged Care in a hybrid format. The core functions of the CEEU include secretariat support for the HTA Consumer Consultative Committee (CCC), which comprises consumer members from all HTA committees. The CEEU also summarizes consumer input for the HTA committees. In 2022, the CEEU launched the Conversations for Change series, which was developed through a range of different consultation activities. As a result, an ongoing “Changing Conversations” mandate is in place, to build and sustain relational, non-transactional partnerships in a true collaborative style.

**Results:**

The HTA committees and subcommittees operate across cycles with overlapping timelines, requiring consumer input to be gathered at different timepoints for different purposes. The CEEU has launched materials, including the Consultation Hub and HTA Engage, and is developing a Consultation Toolkit to raise awareness and facilitate these processes. The role of the HTA CCC is evolving as a forum to enable consumer committee members to share experiences and receive CEEU support. Improvements in consultation processes have enabled the CEEU to provide consumer comments to consumer committee members to facilitate genuine representation of consumer and other external stakeholder views.

**Conclusions:**

Managing consumer input can be resource intensive, however there is increasing recognition of the importance of consumer engagement in Australian HTA. The CEEU expects continued growth and a wider remit from 2024, as new processes and changes occur. Additional materials are planned to support consumer committee members and to build capacity among consumers and consumer organizations.

